# Neonatal and maternal upregulation of antileukoproteinase in horses

**DOI:** 10.3389/fimmu.2024.1395030

**Published:** 2024-04-26

**Authors:** Camille M. Holmes, Susanna Babasyan, Bettina Wagner

**Affiliations:** Department of Population Medicine and Diagnostic Sciences, College of Veterinary Medicine, Cornell University, Ithaca, NY, United States

**Keywords:** innate immunity, reproduction, parturition, neonatal, development, equine, monoclonal antibodies, assay development

## Abstract

**Introduction:**

The end of gestation, ensuing parturition, and the neonatal period represent highly dynamic phases for immunological changes in both mother and offspring. The regulation of innate immune cells at the maternal-fetal interface during late term pregnancy, after birth, and during microbial colonization of the neonatal gut and other mucosal surfaces, is crucial for controlling inflammation and maintaining homeostasis. Innate immune cells and mucosal epithelial cells express antileukoproteinase (SLPI), which has anti-inflammatory and anti-protease activity that can regulate cellular activation.

**Methods:**

Here, we developed and validated new monoclonal antibodies (mAbs) to characterize SLPI for the first time in horses. Peripheral blood and mucosal samples were collected from healthy adults horses and a cohort of mares and their foals directly following parturition to assess this crucial stage.

**Results:**

First, we defined the cell types producing SLPI in peripheral blood by flow cytometry, highlighting the neutrophils and a subset of the CD14+ monocytes as SLPI secreting immune cells. A fluorescent bead-based assay was developed with the new SLPI mAbs and used to establish baseline concentrations for secreted SLPI in serum and secretion samples from mucosal surfaces, including saliva, nasal secretion, colostrum, and milk. This demonstrated constitutive secretion of SLPI in a variety of equine tissues, including high colostrum concentrations. Using immunofluorescence, we identified production of SLPI in mucosal tissue. Finally, longitudinal sampling of clinically healthy mares and foals allowed monitoring of serum SLPI concentrations. In neonates and postpartum mares, SLPI peaked on the day of parturition, with mares returning to the adult normal within a week and foals maintaining significantly higher SLPI secretion until three months of age.

**Conclusion:**

This demonstrated a physiological systemic change in SLPI in both mares and their foals, particularly at the time around birth, likely contributing to the regulation of innate immune responses during this critical period.

## 1 Introduction

Antileukoproteinase (SLPI) is a pleiotropic protein produced systemically and at mucosal surfaces, where it acts to maintain homeostasis. In mammals, it can be detected in the upper (URT) and lower respiratory tract (LRT) (1), female genital tract (FRT) (2), digestive tract (3), parotid glands (4), and breast (5). SLPI structurally resembles a ‘boomerang’, with two physically separated domains on each arm, enabling a range of functionality (6). SLPI can act as either a secreted molecule in the extracellular environment or intracellularly within both the cytosol and nucleus. The most explored properties of SLPI are modulation of inflammation, inhibition of serine proteases, and antimicrobial action. This diverse localization and range of roles establish SLPI as an important regulator in a variety of diseases.

SLPI limits host-driven damage to mucosal barriers, both through the downregulation of inflammatory pathways and the inhibition of proteases. The anti-inflammatory action results from suppression of the NFκB pathway via three mechanisms. First, it can inhibit sCD14 interactions with LPS extracellularly, limiting the responsiveness of cells to LPS (7). Second, it can interrupt the NFκB signaling cascade by blocking the degradation of IκB, preventing NFκB translocation into the nucleus (8). Third, within the nucleus, it competes for the p65 binding site of NFκB stopping the transcription factor from acting (9). Additionally, the C-terminal domain of SLPI is responsible for the inhibition of serine proteases (10) and is independent of its ability to suppress NFκB (11). Moreover, SLPI is one of the primary inhibitors of neutrophil elastase (NE) produced at the mucosa and regulates 80-90% of NE within the respiratory tract by reversible binding (1, 12). Aside from NE, it also demonstrates an affinity for other serine proteases found at the mucosal surfaces including chymotrypsin, cathepsin G, and trypsin (13).

In addition to its role in modulating host-driven processes, SLPI also demonstrates antibacterial, antifungal, and antiviral properties. Through the N-terminal domain, mucosal SLPI is capable of directly killing some bacterial species by interruption of the bacterial cell wall or the inhibition of bacterial nucleic acid replication and transcription (12, 14). Several bacteria, including *Streptococcus pyogenes*, have in turn developed defense mechanisms, such as the production of proteases that degrade SLPI (15). Similarly, antifungal properties are mediated through the N-terminus of SLPI and have been reported to act against *Aspergillus fumigatus* and *Candida albicans* (16). Studies in *C. albicans* also discovered a mechanism of cell membrane interruption, preventing normal fungal growth (17). The antiviral function of SLPI has been most thoroughly explored in human immunodeficiency virus (HIV) and human papillomavirus (HPV). During HIV infection, SLPI can prevent viral entry into host cells by inhibiting HIV interactions with its primary T cell entry receptor CD4 (18). In HPV-infected individuals, SLPI prevents interactions with annexin, which is an entry receptor for the virus (19, 20). Together, SLPI can act on a variety of pathogens by different mechanisms that are independent of its antiprotease and anti-inflammatory roles.

In horses, several transcriptomic studies have demonstrated a conditional upregulation of *SLPI* expression in the reproductive tract (21–25) and exercise-induced disease (26, 27). In the equine endometrium *SLPI* mRNA expression varies depending on gestational stages (21), and there is an upregulation of *SLPI* in endometrial biopsies after experimentally induced endometritis (23–25). *SLPI* mRNA expression in endometrial biopsies also differed with phases of the estrous cycle with higher *SLPI* expression in diestrus (23). Uterine lavage proteomic analysis confirmed protein expression only in diestrus (28). Notably, this trend is contrary to findings in humans, with higher SLPI secretion detected during ovulation and estrus (29). Additionally, racehorses with mild to moderate equine asthma (MMEA) (26) and recurrent exertional rhabdomyolysis (RER) (27), had mRNA upregulation of *SLPI* in the airways and gluteal muscle respectively, compared to healthy controls. Together these reports emphasize the importance of SLPI in equine reproduction and inflammatory conditions. However, the functional roles of SLPI have not yet been characterized in horses because specific antibodies to equine SLPI were not yet available.

In this study, we developed the first monoclonal antibodies (mAbs) to equine SLPI. With these new tools, we characterized leukocyte production of SLPI and quantified systemic and mucosal SLPI normal ranges for the first time in horses. In addition, systemic SLPI concentration was determined in a cohort of healthy mares from parturition through eight months postpartum and their foals until eight months of age.

## 2 Materials and methods

### 2.1 Cloning of the equine SLPI gene

Equine lung tissue was homogenized, and RNA was extracted using the RNeasy kit (Qiagen, Germantown MD, USA). RNA was transcribed into cDNA using the SuperScript Vilo VI kit (Thermo Fisher Scientific, Waltham, MA, USA). First, the whole coding region of equine *SLPI* (GenBank accession number XM_005604653; bases 70-520; forward primer, 5’ TCTCACCATGAAGTCCAGCAG-3’; reverse primer, 5’ TCAGATCAGCGCTTCAATATAGG – 3’) was amplified. Next, equine *SLPI* was cloned into a mammalian expression vector containing an equine interleukin-4 (*IL-4*) gene (IL-4/pcDNA3.1) (30) using primers that included restriction enzyme sites for in-frame cloning of the *SLPI* gene with the 5’ equine *IL-4* gene (forward primer with *BamH I* restriction site, AAGGATCCTGCTAGAAATGCTTCAAAAG; reverse primer with Kpn I restriction site; AAAGGTACCTCAGATCAGCGCTTCAATAT). The same approach was used for equine granulysin (*GNLY)*. RNA was isolated from peripheral blood mononuclear cells (PBMC). The coding region was amplified (GenBank accession number NM_001081929.3; bases 31-480; forward primer, 5’ TGCCCCACCATGACCTCC -3’; reverse primer, 5’ GGCTCAGATGAGACCTGCTT -3’) and then cloned into IL-4/pcDNA3.1 (forward primer with *BamH I* restriction site, AAGGATCCTGGTTTGAACCCTGAGAGCT; reverse primer with Kpn I restriction site, AAAGGTACCTCAGATGAGACCTGCTTTATGTT). The resulting plasmids were Sanger sequenced (Cornell Institute of Biotechnology) and were homologous to the predicted sequences. In this expression system, the IL-4 gene includes a leader sequence promoting the secretion of recombinant protein from transfected cells and serves as a tag for detection of protein expression and protein purification.

### 2.2 Expression of recombinant equine SLPI

Recombinant equine IL-4/SLPI fusion protein (rIL-4/SLPI) was expressed in mammalian cells. Chinese hamster ovary (ExpiCHO-S) cells (Thermo Fisher Scientific, Waltham, MA, USA) were transfected with the IL-4/SLPI/pcDNA3.1 vector, following the manufacturer’s instructions. After 6 days, a cell and serum-free ExpiCHO cell supernatant was collected and rIL-4/SLPI was purified over an anti-equine IL-4 HiTrap column (GE Healthcare, Piscataway, NJ, USA) using an AKTA fast protein liquid chromatography (FPLC) instrument (GE Healthcare, Piscataway, NJ, USA) as previously described (30–32). Purified rIL-4/SLPI protein was separated on a 12% reducing SDS polyacrylamide gel (Bio-Rad, Hercules, CA, USA) and stained with Coomassie Brilliant Blue Dye (Sigma-Aldrich, St. Lois MO, USA). The molecular weight of the rIL-4/SLPI protein was confirmed using a PageRuler Prestained Protein Ladder (10-180 kDa; Thermo Fisher Scientific, Waltham, MA, USA) and protein concentration was determined by bicinchoninic acid assay (Pierce BCA, Thermo Fisher Scientific, Waltham, MA, USA). Additionally, the same transfection protocol was utilized for producing rIL-4/SLPI secreting ExpiCHO-S cells for testing of the SLPI mAbs but transfected cells were harvested after 24 hours of incubation. Another IL-4 fusion protein containing equine granulysin (rIL-4/GNLY) was also produced during this step for testing of SLPI mAb specificity. For the latter procedures, the cells were washed once in phosphate buffered saline (PBS, 137 mM NaCl, 2.7 mM KCl, 4.3 mM Na2HPO), fixed in 2% formaldehyde for 20 minutes at room temperature, and washed twice in PBS. Formaldehyde-fixed transfectants were then resuspended in freezing media (ExpiCHO Expression Media (Thermo Fisher Scientific, Waltham, MA, USA) with 10% DMSO (Sigma-Aldrich, St. Louis, MO, USA)) and stored at -80°C until use.

### 2.3 Development of mAbs against SLPI

Equine-specific mAbs were developed as previously described (30–35). rIL-4/SLPI with 100μL of GERBU adjuvant (GERBU Biotechnik, Heidelberg, Germany) was used for intraperitoneal immunization of a female Balb/C mouse (Jackson Laboratories, Bar Harbor, ME, USA). The first immunization consisted of 100μg rIL-4/SLPI (day 0) followed by several immunizations with 50ug per day (days 14, 21, 35, 49, 70, 91, 112). Finally, the mouse received injections of 50μg rIL-4/SLPI without adjuvant on three consecutive days (days 133, 134, 135), and was euthanized on day 136. Mouse splenocytes were collected and fused with the murine myeloma cell line, X68Ag8.658 (36), to generate hybridoma cell lines secreting mAbs. The mouse procedures were approved by the Institutional Animal Care and Use Committee at Cornell University (protocol 2007−0079).

An ELISA was used to identify SLPI-specific mAbs in cell-free hybridoma supernatants. ELISA wells were coated with a polyclonal anti-equine IL-4 Ab (R&D Systems, Minneapolis, MN, USA) in carbonate coating buffer (1 M NaHCO3, 1 M Na2CO3, [pH 9.6]) overnight at 4°C. The next day, the coating buffer was removed, and wells were blocked with PBS containing 1% BSA. Between each of the following steps, wells were incubated for 1 hour and then washed five times with PBS-T (PBS supplemented with 0.1% Tween 20 (PBS-T, VWR, Radnor, PA, USA)). Next, wells were incubated with purified rIL-4/SLPI at 0.2μg/ml in PBS-T. Then, hybridoma clone supernatants were added. Finally, the binding of mAbs was detected using a peroxidase-conjugated goat anti-mouse IgG(H+L) antibody (Jackson ImmunoResearch Laboratories, West Grove, PA, USA). Positive mAbs were then tested by ELISA against rIL-4/IgG1 (33) to identify IL-4 recognizing mAbs, as previously described (34).

Hybridoma supernatants recognizing SLPI by ELISA were further tested using ExpiCHO transfectants by intracellular staining and flow cytometry. Cells transfected with IL-4/SLPI and IL-4/GNLY were stained in parallel to identify SLPI-specific mAbs. During each step, cells were incubated for 20 minutes, and between steps cells were washed twice with saponin buffer (PBS containing 0.5% saponin, 0.5% BSA, and 0.02% NaN3). First, hybridoma supernatants were diluted 1:1 in saponin buffer and incubated with transfected cells. Next, goat anti-mouse IgG(H+L) F(ab′)2 conjugated to Alexa Fluor 647 (Jackson ImmunoResearch Laboratories, West Grove, PA, USA) in saponin buffer was added. Finally, cells were resuspended in PBS/BSA and were read in a FACSCanto II flow cytometer (BD Biosciences, San Diego, CA, USA). A minimum of 10,000 events per sample were recorded and flow cytometry data was analyzed using FlowJo version 10.8.1 (FlowJo, Ashland, OR, USA).

SLPI-specific mAb clones were transferred and grown up as previously described (37), then the isotypes of each mAb was determined using mouse isotype-specific antibodies (Sigma-Aldrich, St. Louis, MO, USA). Three SLPI mAbs were further characterized, mAb 27-1, 137-1, and 200-3. These mAb clones were adapted to serum-free medium (Hyb-SFM, Thermo Fisher Scientific, Waltham, MA, USA). The mAbs were purified from the supernatants using a HiTrap Protein G HP column (GE Healthcare, Piscataway, NJ, USA) by FPLC as previously described (35). Purified mAbs were then conjugated to Alexa Fluor A488 or A647 (Jackson ImmunoResearch Laboratories, West Grove, PA, USA), or biotin according to manufacturer’s instructions.

### 2.4 Horses

Different samples were collected from Cornell University’s herd of Icelandic horses. Information on horses used for sample collection is summarized in **Table 1**. Horses included stallions, geldings, and mares ranging in age from newborn to 14 years. All horses were housed together on grass pastures with free access to run-in sheds, water, and salt blocks and were fed grass hay in the winter. Healthy horses over the age of one year were used for the initial characterization of systemic and mucosal production. Additionally, mare and foal pairs from the same year were used to characterize SLPI secretion. All mares and foals were healthy throughout the study period. All horse procedures were approved by the Institutional Animal Care and Use Committee at Cornell University (protocol #2011−0011). Additionally, they were carried out by the recommendation in the Guide for the Care and Use of Laboratory Animals of the National Institutes of Health and Guide for Care and Use of Animals in Agricultural Research and Teaching.

**Table 1:** Horse groups, sample types and detection ranges of SLPI in healthy horses.

Sample	Number of Horses	Sex	Age, median (range)	Sample dilution ^a^	SLPI, median (range)
Serum ^b^	24	12 mares 12 geldings	4 years (3-4)	1:5	10.0 ng/mL (4.5-29.1)
Nasal Secretions ^b^	24	12 mares 12 geldings	4 years (3-4)	1:25	464.6 ng/mL (77.9-1090)
Saliva ^b^	16	12 mares 4 geldings	9 years (4 -18)	1:5	31.8 ng/mL (6.3-417.0)
Colostrum ^b^	8	8 mares	6 years (4 -14)	1:25	2065 ng/mL (920.6-5080)
Milk ^b,c^	17	17 mares	6 years (4 -14)	1:10	346.7 ng/mL (93.0-1680)
Serum, mare (within 6 hrs postpartum) ^b^				1:5	2232 ng/mL (482.5-21300)
Serum, mare (2 days postpartum) ^b^				1:5	2231 ng/mL (194.3-1273.1)
Serum, neonatal foal (within 6 hrs after birth) ^b^	17	5 fillies 12 colts	0 days (0)	1:5	757.3 (383.0-9509.2)
Serum, neonatal foal (2 days old) ^b^			2 days (2)		670.1 ng/mL (292.3-1316.4)
Serum, neonatal foal (6 days old) ^b^			6 days (6)		241.7 ng/mL (162.9-434.1)
Serum, foal (10 days old) ^b^			10 days (10)		158.5 ng/mL (71.7-245.2)
Serum, foal (14 days old)			14 days (14)		154.2 ng/mL (65.4-287.4)
Serum, foal (1 month old) ^b^			1 month (1)		43.3 ng/mL (22.6-207.3)
Serum, foal (2 months old) ^b^			2 months (2)		42.6 ng/mL (18.8-147.9)
PBMC ^d^	6	2 mares 3 geldings 1 stallion	11 years (4-18)	n.a.	1.5% (0.3-3.8)
PBL ^d^	6	3 mares 3 geldings	11 years (10-12)	n.a.	54.6% (47.8-65.3)

a All samples were diluted in PBN.

b Samples were quantified in a SLPI fluorescent bead-based assay using rIL-4/SLPI as standard.

c Milk was collected from mares 14 days postpartum.

d Samples from healthy horses were analyzed by flow cytometry and total SLPI^+^ cells were quantified ex vivo.

n.a., not applicable

### 2.5 Peripheral blood collection and processing

Serum and heparinized blood were collected by venipuncture of the jugular vein using a vacutainer system (Becton Dickinson, Franklin Lakes, NJ, USA). The serum was aliquoted and frozen at -20°C. Heparinized blood was allowed to settle at room temperature for 30 minutes to separate erythrocytes from cell-rich plasma and then used to isolate either peripheral blood leukocytes (PBL) or peripheral blood mononuclear cells (PBMC). PBL were isolated from cell-rich plasma by centrifugation at 500 xg for 10 minutes, then washed twice in PBS. PBMC were isolated by density gradient centrifugation (Ficoll-Paque PLUS; GE Healthcare, Piscataway, NJ, USA). Following both isolation procedures, PBL and PBMC were fixed in formaldehyde as described above for ExpiCHO transfectants and stored in PBS/BSA at 4°C until staining and flow cytometric analysis.

### 2.6 Colostrum collection and processing

Colostrum and milk were collected from both the left and right teat of mares into a sterile specimen cup. Colostrum was collected within 6 hours of birth, and milk 14 days postpartum. Samples were aliquoted by avoiding any debris and stored at -20°C. After thawing, the samples were centrifuged for 3 minutes at 300 xg to separate the fat and aqueous phase. The sample added to the assay was taken from the middle of the aqueous phase excluding the milk fat.

### 2.7 Nasal secretion collection and processing

Nasopharyngeal swabs were taken using two cotton-tipped applicators (Puritan Medical Products Company, Gullford, ME, USA). Swabs were directly transferred into a tube containing 2mL of sterile PBS. Samples were maintained at 4°C until processing within 2 hours of collection. Samples were vortexed for 15 seconds and spun at 900 xg for 10 minutes. The aqueous phase was collected and transferred into a fresh tube, centrifuged again for 5 minutes at 300 xg to pellet remaining cells, and the protein containing supernatant was collected and frozen at -20°C until further analysis.

### 2.8 Saliva collection and processing

Saliva was obtained from the horse’s mouth using a spiral bit (kindly provided by Dr. Sigurbjorg Torsteinsdóttir, University of Iceland, Keldur, Iceland) (38), containing absorbent cotton. Horses were allowed to move freely in their stalls for half an hour without access to forage. Cotton was extracted from the spiral bit using forceps and placed into a conical tube containing an unfiltered P1000 pipette tip (TipOne, USA Scientific, Ocala FL, USA). Tubes were centrifuged at 900 xg for 10 minutes to extract saliva from the cotton. The cotton and pipette tip were removed from the tube. Tubes were centrifuged at 900 xg for an additional 10 minutes to pellet debris, and the aqueous phase was collected and stored at -20°C.

### 2.9 Flow cytometry and cell characterization

Intracellular staining of fixed PBL and PBMC was performed with SLPI mAb 27-1 conjugated to Alexa Fluor 647 and diluted in saponin buffer. A murine IgG1 antibody was used as an isotype control. Cells were double or triple-stained with Alexa Fluor 488 conjugated and biotinylated mAbs for equine cell surface markers to characterize the SLPI producing cell populations. PBMC were stained with mAbs against SLPI, CD4 (HB61A) (39) and CD8 (CVS8) (40), SLPI, IgG1 (CVS45) (41), and IgM (1-22) (42), or SLPI and CD14 (105) (43). PBL were stained using mAbs against SLPI, CD14, and CD172a (DH59B). Cells were incubated for 20 minutes with antibody mixes, then washed twice with saponin buffer, before an additional 20-minute incubation with Streptavidin-phycoerythrin (SAV-PE; Invitrogen, Carlsbad, CA, USA) to bind to biotinylated antibodies. A total of 100,000 cells per sample were recorded in a FACSCanto II flow cytometer (BD Biosciences, San Diego, CA, USA). Flow cytometry data was analyzed on FlowJo version 10.8.1 (FlowJo, Ashland, OR, USA).

### 2.10 Fluorescent bead-based assay

For the fluorescent bead-based assay, a total of 100ug SLPI mAb 200-3 was coupled to 5 x10^6^ beads of bead 35 (MicroPlex Microspheres, Luminex, Austin, TX, USA). The assay was performed as previously described for soluble equine proteins (31, 42, 44, 45). Optimal dilutions for sample types were determined (**Table 1**) and all samples were diluted in PBN (PBS containing 1% BSA and 0.05% Sodium Azide, pH 7.4). All samples and reagents were added in 50uL volumes, and incubation of the assay plates (Merckmillipore, Darmstadt, Germany) was performed at room temperature for 30 minutes on a shaker without direct exposure to light. Beads were washed with PBS-T between each step. First, a dilution of the rIL-4/SLPI standard ranging from 50,000 – 76.2 pg/ml, the diluted samples, or PBN for background control were added to the assay plates. Then, 5x10^3^ mAb coupled beads were added to each well and incubated. Next, beads were incubated with biotinylated SLPI mAb 27-1 for detection, and finally with SAV-PE (Invitrogen, Carlsbad, CA, USA). Beads were resuspended in PBN with shaking for 10 minutes and read on a BioPlex200 instrument (Luminex, Austin, TX, USA) and data was processed by BioPlex Manager 6.1 software and reported as concentration in ng/ml.

Additionally, the above assay was used for specificity testing with a variety of other equine proteins. For this test, a panel of previously developed recombinant equine cytokine and chemokine fusion proteins was tested, which included rIL-4/IL-1β, rIL-4/IgG1, rIL-10/IgG4, rIFN-α/IgG4, rIL-4/TNF-α, rIL-4/CCL2, rIL-4/CCL3, rIL-4/CCL5, rCD14/IgG1 (30, 31, 42, 44, 45).

### 2.11 Immunofluorescence of tissues

URT tissue was collected from an adult gelding. Samples were transferred to cold PBS for transport, then were washed in 5 mL PBS and frozen at -80°C in Optimal Cutting Temperature compound (Tissue-Tek OCT, Sakura Finetek USA, Inc. Torrance, CA USA) until tissue staining with SLPI mAb 27-1. Slides were cut from OCT blocks using a cryostat, and 8μm sections were adhered to charged glass slides (Globe Scientific Inc., Mahwah, NJ USA). To prepare for staining, slides were fixed in 10% (v/v) formalin solution (VWR, Radnor, PA USA) for 10 minutes at room temperature and then washed twice in deionized (DI) water. Next, slides were blocked using PBS-T with 10% FCS (Atlanta Biologicals, Flowery Branch, GA, USA) for 1 hour. Tissues were stained overnight at 4°C with 10μg/ml mAb 27-1 in PBS-T with 10% FCS. The primary stain solution was removed, and tissues were washed twice with PBS-T. Next, tissues were stained for 1 hour at room temperature with a fluorescent secondary antibody (Goat anti-Mouse IgG (H+L) Cross-Adsorbed Secondary Antibody, Alexa594; Thermo Fisher Scientific, Waltham, MA, USA). Finally, secondary stain solution was removed, and tissues were washed twice with PBS-T, followed by DI water. Slides were mounted with DAPI (SlowFade Diamond Antifade Mountant with DAPI, Thermo Fisher Scientific, Waltham, MA, USA) and stored in the dark at 4°C until same day visualization on a Leica DM2500 upright microscope with a DFC7000T camera for fluorescence and bright field microscopy (Leica Microsystems, Wetzlar, Germany). SLPI was visualized at 594 nm and DAPI was visualized at 359 nm. Images from both wavelengths were merged using Leica Application Suite X (v1.4.5, Leica Microsystems, Wetzlar, Germany).

### 2.12 Statistical analysis

All statistical analysis was performed using GraphPad Prism software version 8 (GraphPad Software, La Jolla, CA, USA). Data sets were tested by Shapiro-Wilks normality tests, were not normally distributed, and further analyzed using non-Gaussian statistical tests. Mucosal and systemic SLPI concentrations were compared by Kruskal-Wallis tests with Dunn’s multiple comparisons. Secretion in milk vs. colostrum was compared by Wilcoxon matched pairs signed rank test. Mare and foal serum secretion of SLPI was compared by a Mixed-effect model with Giesser-Greenhouse correction and Bonferroni’s multiple comparisons tests. Foal Serum SLPI concentrations on day 2 and mare colostrum SLPI concentrations were calculated by a Spearman’s rank correlation. P-values <0.05 were considered significant. All graphs were made with GraphPad Prism software version 8.

## 3 Results

### 3.1 Development of equine SLPI mAbs

**Figure 1:**
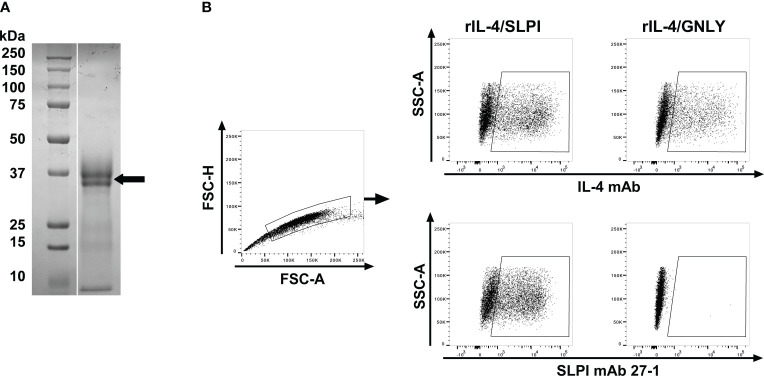
Expression of a recombinant equine IL-4/SLPI fusion protein and flow cytometric analysis of transfectants after intracellular staining with mAbs against equine SLPI. **(A)** Recombinant equine IL-4/SLPI was expressed in mammalian cells and purified from the cell culture supernatant. Purified rIL-4/SLPI protein was separated on an SDS-gel and stained by Coomassie Brilliant Blue (right lane), with a protein size marker shown in the left lane. The protein representing rIL-4/SLPI is marked by an arrow. Equine SLPI mAbs were then developed by immunization of a Balb/C mouse with rIL-4/SLPI. **(B)** SPLI mAbs were used for intracellular staining of ExpiCHO transfectants expressing IL-4/SPLI or IL-4/GNLY fusion proteins. Cells were analyzed by flow cytometry. A gate was set by forward scatter (FSC) height and area for singlets. IL-4/SLPI or IL-4/GNLY transfectants were stained for intracellular expression with either IL-4 mAb 13G7 (upper panel) or SLPI mAb 27-1 (lower panel).

To investigate SLPI in horses, mAbs against equine SLPI were developed using hybridoma technology after immunization of mice with purified rIL-4/SLPI. The relative molecular weight of the recombinant fusion protein, excluding glycosylation, was calculated as 30.97 kDa. Its size was determined as approximately 30 kDa by SDS-PAGE, with two closely sized protein bands likely representing different glycosylation forms (**Figure 1A**). After cell fusion, hybridoma supernatants were tested against rIL-4/SLPI and rIL-4/IgG1 by ELISA. The rIL-4/IgG1 assay was performed to exclude mAbs directed against the IL-4 fusion partner. Three SLPI mAbs were identified, 27-1, 137-1 and 200-3, and characterized in more detail. SLPI positive supernatants were next tested by intracellular staining of IL-4/SLPI and IL-4/GNLY transfected ExpiCHO cells and flow cytometric analysis (**Figure 1B**). SLPI mAb 27-1 and a mAb for equine IL-4 recognized a similar population of IL-4/SLPI transfected cells. However, the IL-4/GNLY transfectant was only recognized by the IL-4 mAb and not by SLPI mAb 27-1. The other SLPI mAbs, 137-1 and 200-3, had similar staining pattern with these transfectants. Together this demonstrated the detection of recombinant equine SLPI by the three SPLI mAbs.

### 3.2 Characterization of SLPI-producing leukocytes in peripheral blood

**Figure 2:**
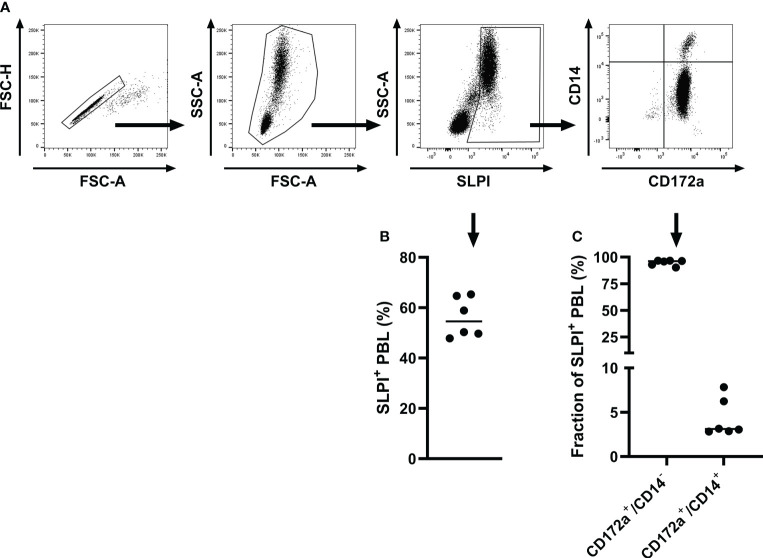
Intracellular staining and flow cytometric analysis of equine PBL with SLPI mAb 27-1. PBL were isolated from healthy adult horses (n=6) and analyzed for production of SLPI by flow cytometry. **(A)** A representative gating strategy for the analysis of SLPI^+^ cells is shown for one horse. Cells were gated on singlets, then on leukocytes, then on SLPI^+^ cells, and finally multicolor staining for cell surface markers was used to further define SPLI^+^ cell types based on CD14 and CD172a expression. **(B)** The percentages of SLPI^+^ cells within the total PBL population were measured in six horses. **(C)** Quantification of CD172a^+^/CD14^-^ and CD172a^+^/CD14^+^ cells within the total SLPI^+^ population. Individual dots in **(B, C)** represent single horses and the horizontal bars show the medians.

Next, the SLPI mAbs were evaluated by intracellular staining of equine PBL to confirm recognition of native SLPI. PBL were isolated, fixed, and stained intracellularly with SLPI mAbs. Flow cytometric analysis revealed constitutive SLPI expression in equine PBL ex vivo (**Figure 2**). Based on forward and side scatter characteristics, the majority of SLPI^+^ cells were large, complex, and located in the neutrophil scatter (**Figure 2A**). In total 56.1 ± 7.9% of the leukocytes were positive for SLPI production using mAb 27-1 (**Figure 2B**). Cell surface marker staining further characterized the SLPI^+^ population: all SLPI^+^ cells were positive for CD172a, a marker that is expressed on equine neutrophils and on other cell types. Staining with the monocyte marker CD14 separated SLPI+/CD172a^+^ cells into two populations resulting in a major CD14^-^/CD172a^+^ neutrophil (94.8 ± 2.6%) and a smaller CD14^+^/CD172a^+^ monocyte population (4.3 ± 2.2%) (**Figure 2C**).

**Figure 3:**
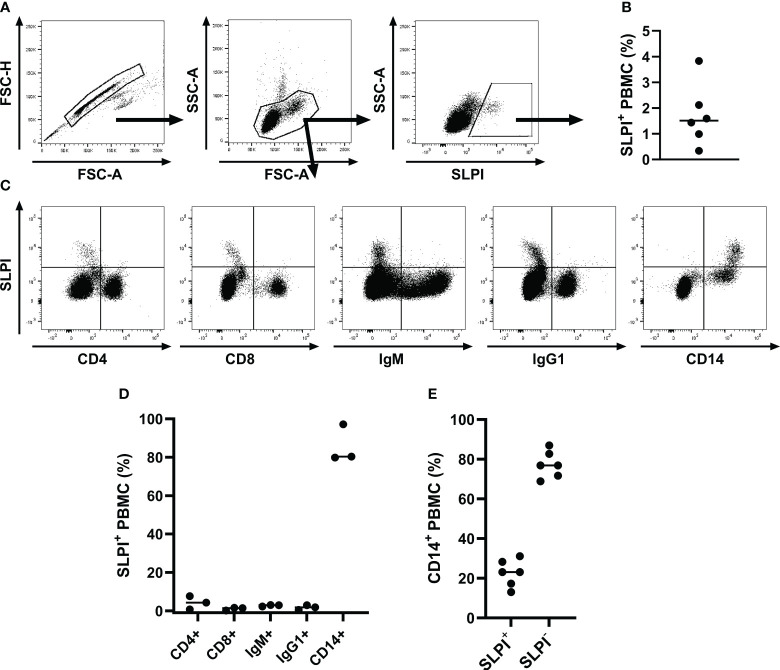
Flow cytometric analysis of SLPI producing PBMC. PBMC were isolated from healthy horses (n=6), fixed, stained intracellularly with SLPI mAb 27-1, and analyzed by flow cytometry. **(A)** A representative gating strategy for SLPI^+^ cells in one horse is shown. First, a gate was placed on singlets, then on PBMC, and finally on SLPI^+^ cells. **(B)** The percentages of SLPI^+^ cells within the total PBMC population are shown. **(C)** Multicolor staining for cell surfaces markers was used to define SLPI^+^ cell types in PBMC. Representative examples from PBMC of one horse stained for SLPI and CD4, CD8, IgM, IgG1, and CD14. **(D)** The percentages of each cell type within the total SLPI^+^ population were quantified in three horses. **(E)** The percentages of SLPI^+^ cells within the total CD14^+^ cell population were measured in six horses. **(B, D, E)** Dots represent individual horses and horizontal lines represent medians.

Additionally, intracellular staining was performed with PBMC to further evaluate mononuclear cell types contributing to SLPI production (**Figure 3**). A small population of SLPI^+^ cells (1.7 ± 1.2%) was identified in PBMC (**Figures 3A, B**). CD4^+^ and CD8^+^ T cells and IgM^+^ and IgG1^+^ B cells all lacked expression of SLPI (**Figure 3C**). In agreement with results from PBL, most SLPI expressing cells were CD14^+^ (**Figures 3C, D**). Within the total CD14^+^ population, 22.7 ± 6.7% of cells were SLPI^+^ (**Figure 3E**). The other two SLPI mAbs recognized a similar population within PBMC (**Table 2**). **Supplementary Figure S1** shows the comparison of all three SLPI mAbs for intracellular staining of PBMC.

**Table 2:** Applications of three novel mAbs against equine SLPI.

	Flow Cytometry	Bead-based Assay	Immunofluorescence
mAb 27-1	+++	+++^a^	+++
mAb 137-1	+	++	n.t.
mAb 200-3	+	+++^b^	n.t.

a mAb selected for detection.

b mAb selected for bead coupling.

n.t., not tested

+++, highest sensitivity; ++, decreased sensitivity; +, lowest sensitivity.

Together this identified equine neutrophils and a subpopulation of monocytes as the main SLPI producing cells in peripheral blood. SLPI production was evaluated without any additional cell stimulation, emphasizing the constitutive production of SLPI by these two cell types in healthy adult horses.

### 3.3 Development of fluorescent bead-based assay for detection of secreted SLPI

**Figure 4:**
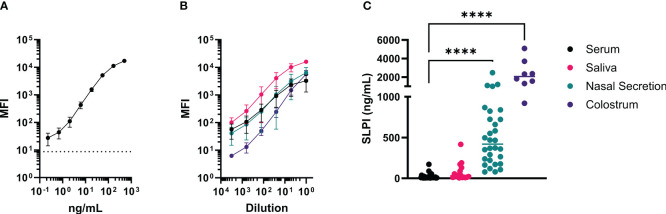
A fluorescent bead-based assay was developed with the new mAbs for quantification of SLPI in various samples from horses. This assay was established using rIL-4/SLPI as a standard for SLPI quantification in different samples and secretions from healthy adult horses. For the assay, mAb 200-3 was coupled to the beads and SLPI mAb 27-1 was biotinylated for detection. **(A)** The rIL-4/SLPI standard was tested in 12 assays to determine reproducibility and range of detection. The average blank value is displayed as a horizontal dotted line. **(B)** Titration curves of different equine samples including serum (black, n=6), nasal secretions (red, n=6), saliva (teal, n=6), and colostrum (purple, n=4). Each sample was tested undiluted and serial dilution steps of 1:5. **(A, B)** points represent means and error bars represent standard deviations, **(C)** SPLI concentrations were measured in horse serum (n=24), saliva (n=16), nasal secretions (n=24), and colostrum (n=8). Individual points represent individual horses and horizontal lines represent medians. ****p<0.0001.

After initial testing of all possible SLPI mAb pairs (**Supplementary Figure S2**), two mAbs were selected for the SLPI fluorescent bead-based assay: mAb 200-3 was coupled to the beads and mAb 27-1 was used for detection (**Table 2**). The linear detection range of the assay was 230pg/ml-166ng/ml (**Figure 4A**). The specificity of the SLPI assay was confirmed by testing a panel of recombinant equine cytokine and chemokine fusion proteins (**Table 3**). Only rIL-4/SLPI was detected but none of the other cytokines, chemokines, or IgGs that were applied to the assay. Finally, the assay was used to measure SLPI in serum, nasal secretion, saliva, and colostrum samples from healthy horses (**Figure 4C**). In agreement with the expected basal secretion of SLPI systemically and at mucosal surfaces, SLPI was detected in all sample types at various sample-specific baseline concentrations (**Table 1**). Overall, serum and saliva SLPI concentrations were similar in healthy adult horses, while increased SLPI was found in nasal secretions and in the colostrum (both p<0.0001).

**Table 3:** Specificity of the equine SLPI bead-based assay.

Tag	Fusion Protein^a^										
	IL-4						IgG1		IgG4		
**Target protein**	SLPI	IL-1β	TNF-α	CCL2	CCL3	CCL5	IL-4	CD14	IL-10	IFN-α	Buffer
**MFI**^b^	13183	4	4	4	3	2	2	2	3	3	4

a Equine IL-4 or the constant heavy chain regions of equine IgG1 or IgG4 were used as a tag for recognition and purification of the fusion proteins. The IL-4 tag also promotes secretion of recombinant proteins.

b Median fluorescent intensity.

### 3.4 SLPI secreting cells in mucosal tissue

**Figure 5:**
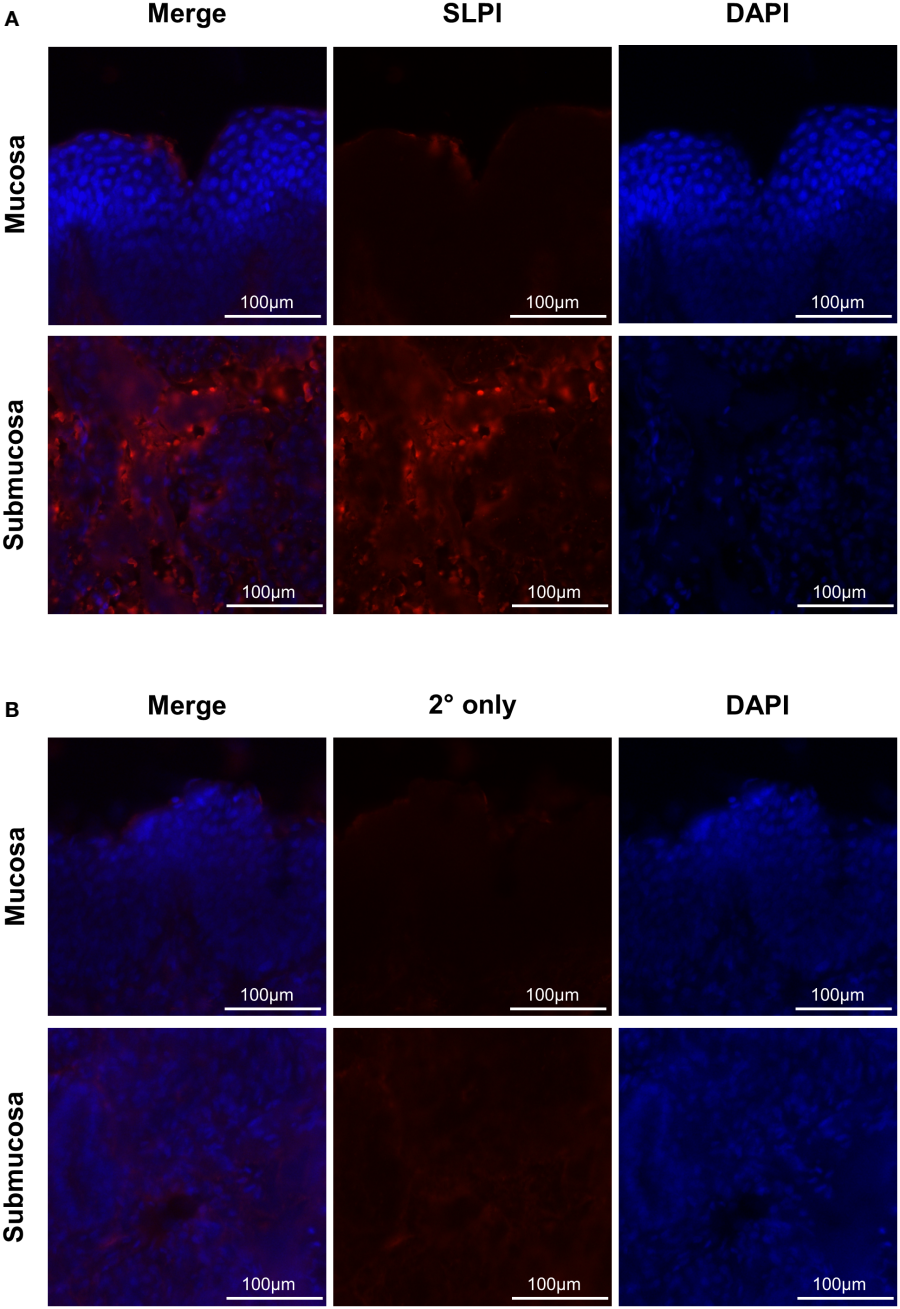
Immunofluorescence staining of SLPI producing cells. To determine localization of mucosal SLPI secretion, nasal tissue samples were collected from a healthy horse and immediately frozen in OCT. Tissues were fixed and stained for SLPI expression (red) and nuclear regions by DAPI (blue). Images were acquired at 595nm for **(A)** SLPI mAb 27-1 or **(B)** secondary only control, 359 nm for DAPI, and merged to form a composite. Displayed are representative images from the mucosa and submucosa (rows), with each channel and a composite image (columns).

In addition, SLPI secreting cells were identified in mucosal tissue of the URT by immunofluorescence (**Table 2**) and SLPI expressing cells were visualized (**Figure 5**). The majority of SLPI production was localized in the submucosa of the nasal tissue, while the epithelial surface showed only minor protein expression. SLPI expression by cells in the nasal submucosa was in agreement with the baseline SLPI concentrations in nasal secretions from healthy horses (**Figure 4C**, **Table 1**).

### 3.5 Maternal SLPI and foal serum SLPI concentrations

**Figure 6:**
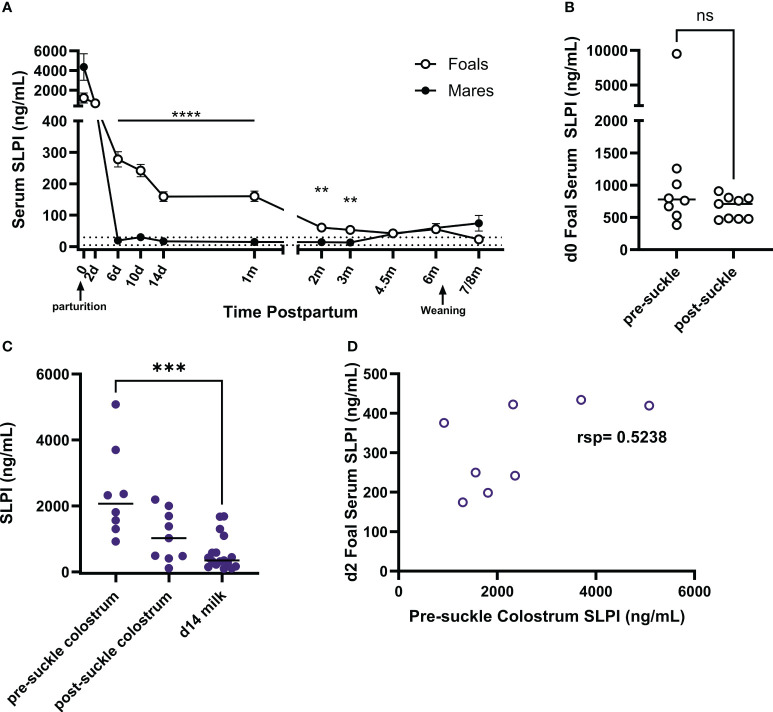
Quantification of SLPI in colostrum and in serum from mares and their foals. SLPI was measured in serum and colostrum samples using the bead-based assay. **(A)** SLPI concentrations were quantified in the serum of mares (n=17) and foals (n=17) from the day of parturition to post-weaning of the foals. The data represent means and standard errors. The dotted lines indicate the normal serum SLPI concentration min-max range in adult horses. **(B)** On day 0, some serum samples were taken before the foals suckled (pre-suckle, n=8) while others were taken post-suckle within 6 hours after birth (n=9). Serum SLPI concentrations of individual foals are represented by dots and horizontal lines are medians. **(C)** SLPI was also quantified in pre- (n=8) and post-suckle (n=9) colostrum and milk (n=17) collected from the dams, with individual dots representing horses and horizontal lines showing medians. **(D)** Foal (n=8) serum SLPI concentrations on day 2 of age were compared to pre-suckle colostrum SLPI concentrations from their dams. rsp, Spearman rank correlation; ** p<0.01, *** p<0.001, ****p<0.0001, ns p>0.05.

High concentrations of SLPI in colostrum supported transmission of SLPI from dams to their newborn foals and suggested potential transfer of maternal SLPI into the foal circulation. SLPI was measured in serum from mares and their foals (n=17). Samples were taken starting within the first six hours after birth (day 0) and at several times until 7-8 months postpartum (pp) (**Figure 6A**). SLPI serum concentrations on day 0 ranged from 482.5 to 21,300 ng/mL in mares (**Table 1**). Mares had significantly higher serum concentrations on days 0 and 2 pp compared to normal SLPI serum values in adult horses (p<0.0001). Then, serum SLPI decreased and reached the normal adult range by day 6 pp.

Foal serum SLPI concentrations on day 0 were between 383.0 to 9,509.2 ng/mL and were similar on day 2. Then, SLPI in foal serum decreased but was still significantly higher than concentrations of their dams on days 6 (p<0.0001), 14 (p<0.0001), 30 (p<0.0001), 60 (p=0.0059), and 90 (p=0.0007) of age. After 4.5 months of age, foal SLPI had declined to the normal adult range. In addition, serum SLPI concentrations were evaluated in neonates that had not yet suckled colostrum (pre-suckle, n=8) and those that had already suckled some colostrum (post-suckle, n=9). The post-suckle samples were all taken within the first 6 hours after birth (**Figure 6B**). SLPI serum concentrations were high before neonates suckled colostrum and comparable between pre- and post-suckling samples.

SLPI concentrations were also measured in the colostrum and milk of the mares. Eight colostrum samples were collected before the foal first suckled (pre-suckle colostrum), while nine samples were collected afterward (post-suckle colostrum) but within the first 6 hours pp. SLPI concentrations were significantly higher (p=0.0007) in pre-suckle colostrum compared to milk on day 14 pp (**Figure 6C**). The correlation of SLPI concentrations in pre-suckle colostrum and foal serum taken on day 2 of age was moderate (**Figure 6D**, rsp=0.5238). Together the data from healthy mares and foals implied that SLPI plays essential roles in mares at the time of foaling and in the early life of foals. The moderate correlation between colostrum and foal serum SLPI may suggest that maternal SLPI can be transferred to the foal’s circulation, but neonatal foals also had high SLPI serum concentrations before colostrum intake supporting an elevated endogenous SLPI production by young foals.  

## 4 Discussion

SLPI is involved in innate immune responses with a broad repertoire of mechanisms to reduce or prevent host- and pathogen-derived damage and maintain homeostasis. It is constitutively secreted systemically and at mucosal surfaces, where it acts as a negative regulator of inflammation (7–9), an inhibitor of serine protease activity (1, 12, 13), and a first-line defender in reproductive and respiratory viral (18–20), bacterial (12–14), and fungal infections (17). A previously described equine neutrophil antimicrobial protein, eNAP-2, has similar antimicrobial and antiprotease activity as human SLPI (46, 47). Past publications by Marth et al. have implied equine SLPI is synonymous with eNAP-2, however, they are two separate and distinct equine proteins, with amino acid homology limited to a common whey acidic protein (WAP) domain (14). Thus, previous assessments of equine SLPI have been limited to RNA expression, which have demonstrated changes in *SLPI* expression in the reproductive tract throughout the estrous cycle and during inflammatory diseases (21–25). In this study, we characterized SLPI in serum, different mucosal secretions, peripheral blood cells and URT mucosa tissue of healthy horses.

We first developed novel mAbs for the detection and quantification of equine SLPI. In humans a commercially available ELISA (Quantikine Immunoassasys, R&D Systems; Wiesbaden-Nordenstadt, Germany) identified median normal SLPI serum values in middle aged adults as 32ng/mL. Using this assay for several clinical studies in human patients focusing on cancer, viral infection, and surgical recovery, the healthy control patients had median SLPI serum concentrations of 32ng/mL (38 women, range 25–43ng/mL) (48), 39.7 ng/mL (17 women, range 33.9-52.8ng/mL) (49), 39.5ng/mL (12 men, range 28.1-46.9ng/mL) (49), and 40.1ng/mL (226 pre-cardiac surgery, 31.6-48.5 ng/mL) (50). Here, we measured SLPI in healthy adult horses which had a median serum SLPI concentrations of 10ng/mL. In the peripheral blood of healthy horses, neutrophils were identified via flow cytometry as the major continuous producers of equine SLPI. In addition, a sub-population of equine monocytes expressed SLPI. Recent characterization of equine PBMC by single cell RNAseq supported the presence of classical, intermediate, and non-classical monocyte subsets in horses, and *SLPI* was expressed in the three clusters identified as classical monocytes but was not expressed in any other cell type described (51). In line with what we found in horses, neutrophil and monocyte production of SLPI has previously been described in humans (52, 53). Additionally, previous work in human and murine cells supported the rapid uptake of exogenous SLPI into immune cells during inflammatory conditions, including B cells and monocytes (8, 9, 54). It is currently unknown whether SLPI uptake is receptor-dependent or -independent. However, it was suggested that SLPI might enter through interactions with cell surface receptors, such as annexin and scramblase, which also contribute to its anti-viral properties (18–20, 55). Further exploration would be needed to determine interactions of exogenous SLPI with immune cells in horses. In addition to immune cells, SLPI production was detected in submucosal cells of the URT from a healthy horse. This suggests that different cell types can produce SLPI and contribute to the baseline that was observed in serum and different samples from healthy horses. In particular, the submucosal region may act as the predominant source of mucosal SLPI under normal conditions. Previous work in humans also supports submucosal expression of SLPI in the URT (56), and studies of the LRT demonstrated submucosal gland expression of *SLPI* mRNA as nearly 30-fold higher than surface epithelium, providing an explanation for increased SLPI secretion in this region (57).

In mares, we observed elevated concentrations of serum SLPI after normal parturition, suggesting a function of SLPI in the changing maternal-fetal immune interface as mares reach the end of gestation. In humans, SLPI in cervical fluids increased throughout pregnancy reaching a peak postpartum (2, 58). Similarly, in vaginal secretions, higher SLPI concentrations were found during pregnancy compared to non-pregnant women (59). However, all previous work has focused on localized changes in SLPI production, and to our knowledge this current data set is the first longitudinal report of SLPI serum concentration changes after parturition. The increase in SLPI around birth could be explained by the occurrence of neutrophilia, which is characteristic of late term gestation and parturition in humans (60–62) and horses (63–66). With an increasing number of neutrophils, a higher concentration of SLPI can be expected, especially in the context of cellular activation and inflammation when neutrophils localize to the reproductive tract in women and help to promote labor (67). Neutrophils have been detected in postpartum equine endometrial samples with decreasing frequency over time (68), suggesting a similar neutrophil localization during parturition. As such, SLPI has been suggested as a marker for pre-term labor in women (58, 69), but it is unknown whether this change in SLPI production and thus the inflammatory environment of the reproductive tract, is associated with pre-term delivery or just a characteristic of delivery itself. In the early days postpartum, SLPI may play an important role in preventing neutrophil derived damage due to the increased numbers present in the mare’s uterus after birth and promote the return to homeostasis following increased intrauterine inflammation associated with parturition. The detailed functions and mechanisms of SLPI around parturition have yet to be explored in future studies.

The mammary gland is a non-classical component of the mucosal immune system promoting passive transfer of immune components from the dam to her foal. In this study, we identified equine colostrum as a significant source of SLPI with concentrations up to 170 times higher than in serum. SLPI has also been detected in the colostrum and breast milk of humans, decreasing with time postpartum (70). Maternal SLPI is likely undergoing proteolytic degradation in the neonatal stomach and duodenum, which has been described in humans following oral SLPI treatments (71, 72). Nevertheless, colostrum- and milk-derived SLPI likely have important anti-microbial effects in the oral mucosa, as previously suggested in infants of HIV^+^ mothers (5, 70, 73). In horses, maternal transfer of immunoglobulins is crucial for neonatal health. Passive transfer of maternal antibodies can be detected in the neonatal foal’s circulation within a few hours after colostrum intake, and colostrum and neonatal foal serum antibody concentrations highly correlate after successful maternal transfer (74–76). For SLPI, the correlation between colostrum and foal serum concentrations was only moderate. We also showed that healthy equine neonates already had high serum SLPI concentrations before they suckled colostrum, and that SLPI had not further increased after completed colostrum intake by 2 days of age. This strongly suggested that maternal SLPI may have important functions at the intestinal mucosa but is not, or only in minor amounts, transferred to the neonatal circulation. The moderate correlation between SLPI in colostrum and neonatal serum could therefore be solely a result of the ability of foals to produce their own SLPI.

Nevertheless, high amounts of endogenous SLPI in the circulation of healthy foals before suckling colostrum and until several months after birth confirmed a vital role for SLPI and a need for its prolonged production during neonatal and young foal development. One potential physiological factor driving elevated SLPI production in neonatal and young foals is early life colonization by environmental microbes, which is crucial for long-term health but also requires immune tolerance rather than strong responsiveness (77). As a mucosal homeostatic regulator, SLPI could be essential for maintaining this balance in young foals. For example, increased SLPI production by dendritic cells in murine mucosal lymph nodes decreased responses to LPS through reduced cellular activation (78). Human infant-derived buccal epithelial cells developed hyporesponsive to bacteria as they upregulated SLPI (79), suggesting SLPI plays a part in promoting tolerance to microbial colonization. Along these lines, dysregulation of SLPI has been associated with clinical conditions that impact mucosal surfaces of human neonates and infants. In premature human neonates requiring manual respiration, the proportion of SLPI to its neutrophil-derived proteases was indicative of the development of bronchopulmonary dysplasia (BDP): neonates who developed BDP had a lower SLPI inhibitory capacity (80, 81) or a lower ratio of SLPI to its protease targets (82). In inflammatory conditions, such as irritable bowel disease (IBD), infants upregulated *SLPI* (83). In mouse models of colitis, the same upregulation of *SLPI* occurred, promoting recovery from disease through inhibition of neutrophil elastase (84, 85). Together, this indicated that mucosal inflammation improves when SLPI and protease activation are balanced at mucosal surfaces in neonates and young individuals. Our data strongly suggested that equine neonates and foals have a similar need for SLPI in regulating inflammation, maintaining homeostasis, and ultimately supporting foal health during the intense phase of exposure to multiple new environmental and antigenic triggers after birth.

## 5 Conclusion

New mAbs recognizing equine SLPI enabled, for the first time, the characterization of this protein in horses. This demonstrated baseline concentrations of SLPI both systemically and at mucosal surfaces in healthy adult horses, and identified neutrophils, CD14^+^ monocytes, and submucosal cells as the cell types contributing to this constitutive production. Further, we evaluated SLPI production in mares after parturition and their foals during neonatal and early life. This demonstrated a significantly heightened maternal SLPI concentration postpartum, both in serum and in the mammary gland. Similarly, foals had high concentrations of SLPI directly following birth, but unlike mares, foal serum values remained significantly elevated compared to adults for the first three months of life. Together this article provides a characterization of the cellular origin of SLPI in horses, baseline SLPI concentrations in various samples, and identifies increased SLPI concentrations after parturition and in early foal life.

## Data availability statement

The original contributions presented in the study are included in the article/**Supplementary Material**. Further inquiries can be directed to the corresponding author.

## Ethics statement

The animal study was approved by Institutional Animal Care and Use Committee at Cornell University. The study was conducted in accordance with the local legislation and institutional requirements.

## Author contributions

CH: Conceptualization, Formal analysis, Investigation, Methodology, Validation, Visualization, Writing – original draft, Writing – review & editing, Data curation. SB: Methodology, Supervision, Writing – review & editing. BW: Conceptualization, Funding acquisition, Investigation, Methodology, Project administration, Resources, Supervision, Writing – original draft, Writing – review & editing.

## References

[1] Lee CH Igarashi Y Hohman RJ Kaulbach H White MV Kaliner MA. Distribution of secretory leukoprotease inhibitor in the human nasal airway. Am Rev Respir Dis. (1993) 147:710–6. doi: 10.1164/ajrccm/147.3.710 8095126

[2] Helmig R Uldbjerg N Ohlsson K. Secretory leukocyte protease inhibitor in the cervical mucus and in the fetal membranes. Eur J Obstetrics Gynecology Reprod Biol. (1995) 59:95–101. doi: 10.1016/0028-2243(94)02023-8 7781866

[3] Franken C Meijer CJ Dijkman JH. Tissue distribution of antileukoprotease and lysozyme in humans. J Histochem Cytochem. (1989) 37:493–8. doi: 10.1177/37.4.2926127 2926127

[4] Thompson RC Ohlsson K. Isolation, properties, and complete amino acid sequence of human secretory leukocyte protease inhibitor, a potent inhibitor of leukocyte elastase. Proc Natl Acad Sci USA. (1986) 83:6692–6. doi: 10.1073/pnas.83.18.6692 PMC386575 3462719

[5] Farquhar C VanCott TC Mbori-Ngacha DA Horani L Bosire RK Kreiss JK. Salivary secretory leukocyte protease inhibitor is associated with reduced transmission of human immunodeficiency virus type 1 through breast milk. J Infect Dis. (2002) 186:1173–6. doi: 10.1086/343805 PMC3382060 12355371

[6] Grütter MG Fendrich G Huber R Bode W. The 2.5 A X-ray crystal structure of the acid-stable proteinase inhibitor from human mucous secretions analysed in its complex with bovine alpha-chymotrypsin. EMBO J. (1988) 7:345–51. doi: 10.1002/embj.1988.7.issue-2 PMC454325 3366116

[7] Ding A Thieblemont N Zhu J Jin F Zhang J Wright S. Secretory leukocyte protease inhibitor interferes with uptake of lipopolysaccharide by macrophages. Moore RN editor. Infect Immun. (1999) 67:4485–9. doi: 10.1128/IAI.67.9.4485-4489.1999 PMC96768 10456890

[8] Taggart CC Greene CM McElvaney NG O’Neill S. Secretory leucoprotease inhibitor prevents lipopolysaccharide-induced IκBα Degradation without affecting phosphorylation or ubiquitination. J Biol Chem. (2002) 277:33648–53. doi: 10.1074/jbc.M203710200 12084717

[9] Taggart CC Cryan SA Weldon S Gibbons A Greene CM Kelly E. Secretory leucoprotease inhibitor binds to NF-κB binding sites in monocytes and inhibits p65 binding. J Exp Med. (2005) 202:1659–68. doi: 10.1084/jem.20050768 PMC2212970 16352738

[10] Eisenberg SP Hale KK Heimdal P Thompson RC. Location of the protease-inhibitory region of secretory leukocyte protease inhibitor. J Biol Chem. (1990) 265:7976–81. doi: 10.1016/S0021-9258(19)39026-X 2110563

[11] Yang J Zhu J Sun D Ding A. Suppression of macrophage responses to bacterial lipopolysaccharide (LPS) by secretory leukocyte protease inhibitor (SLPI) is independent of its anti-protease function. Biochim Biophys Acta (BBA) - Mol Cell Res. (2005) 1745:310–7. doi: 10.1016/j.bbamcr.2005.07.006 16112212

[12] Miller KW Evans RJ Eisenberg SP Thompson RC. Secretory leukocyte protease inhibitor binding to mRNA and DNA as a possible cause of toxicity to Escherichia coli. J Bacteriol. (1989) 171:2166–72. doi: 10.1128/jb.171.4.2166-2172.1989 PMC209873 2467900

[13] Wright CD Kennedy JA Zitnik RJ Kashem MA. Inhibition of murine neutrophil serine proteinases by human and murine secretory leukocyte protease inhibitor. Biochem Biophys Res Commun. (1999) 254:614–7. doi: 10.1006/bbrc.1998.0108 9920787

[14] Hiemstra PS Maassen RJ Stolk J Heinzel-Wieland R Steffens GJ Dijkman JH. Antibacterial activity of antileukoprotease. Infect Immun. (1996) 64:4520–4. doi: 10.1128/iai.64.11.4520-4524.1996 PMC174407 8890201

[15] Draper D Donohoe W Mortimer L Heine RP. Cysteine proteases of *trichomonas vaginalis* degrade secretory leukocyte protease inhibitor. J Infect Dis. (1998) 178:815–9. doi: 10.1086/515366 9728551

[16] Tomee JFC Hiemstra PS Heinzel-Wieland R Kauffman HF. Antileukoprotease: an endogenous protein in the innate mucosal defense against fungi. J Infect Dis. (1997) 176:740–7. doi: 10.1086/514098 9291323

[17] Curvelo JA da R Barreto ALS Portela MB Alviano DS Holandino C Souto-Padrón T. Effect of the secretory leucocyte proteinase inhibitor (SLPI) on Candida albicans biological processes: A therapeutic alternative? Arch Oral Biol. (2014) 59:928–37. doi: 10.1016/j.archoralbio.2014.05.007 24907522

[18] McNeely TB Dealy M Dripps DJ Orenstein JM Eisenberg SP Wahl SM. Secretory leukocyte protease inhibitor: a human saliva protein exhibiting anti-human immunodeficiency virus 1 activity in vitro. J Clin Invest. (1995) 96:456–64. doi: 10.1172/JCI118056 PMC185219 7615818

[19] Skeate JG Porras TB Woodham AW Jang JK Taylor JR Brand HE. Herpes simplex virus downregulation of secretory leukocyte protease inhibitor enhances human papillomavirus type 16 infection. J Gen Virology. (2016) 97:422–34. doi: 10.1099/jgv.0.000341 PMC4804641 26555393

[20] Woodham AW Da Silva DM Skeate JG Raff AB Ambroso MR Brand HE. The S100A10 subunit of the annexin A2 heterotetramer facilitates L2-mediated human papillomavirus infection. PLoS One. (2012) 7:e43519. doi: 10.1371/journal.pone.0043519 22927980 PMC3425544

[21] Badinga L Michel FJ Fields MJ Sharp DC Simmen RCM. Pregnancy-associated endometrial expression of antileukoproteinase gene is correlated with epitheliochorial placentation. Mol Reprod Dev. (1994) 38:357–63. doi: 10.1002/mrd.1080380402 7980943

[22] Khan FA Diel de Amorim M Chenier TS. Qualitative analysis and functional classification of the uterine proteome of mares in oestrus and dioestrus. Reprod Dom Anim. (2020) 55:1511–9. doi: 10.1111/rda.13800 32772405

[23] Marth CD Firestone SM Glenton LY Browning GF Young ND Krekeler N. Oestrous cycle-dependent equine uterine immune response to induced infectious endometritis. Vet Res. (2016) 47:110. doi: 10.1186/s13567-016-0398-x 27825391 PMC5101692

[24] Marth CD Firestone SM Hanlon D Glenton LY Browning GF Young ND. Innate immune genes in persistent mating-induced endometritis in horses. Reprod Fertil Dev. (2018) 30:533. doi: 10.1071/RD17157 28834688

[25] Marth CD Young ND Glenton LY Noden DM Browning GF Krekeler N. Deep sequencing of the uterine immune response to bacteria during the equine oestrous cycle. BMC Genomics. (2015) 16:934. doi: 10.1186/s12864-015-2139-3 26572250 PMC4647707

[26] Karagianni AE Kurian D Cillán-Garcia E Eaton SL Wishart TM Pirie RS. Training associated alterations in equine respiratory immunity using a multiomics comparative approach. Sci Rep. (2022) 12:427. doi: 10.1038/s41598-021-04137-3 35013475 PMC8748960

[27] Valberg SJ Velez-Irizarry D Williams ZJ Henry ML Iglewski H Herrick K. Enriched pathways of calcium regulation, cellular/oxidative stress, inflammation, and cell proliferation characterize gluteal muscle of standardbred horses between episodes of recurrent exertional rhabdomyolysis. Genes. (2022) 13:1853. doi: 10.3390/genes13101853 36292738 PMC9601720

[28] Khan N Hislop A Gudgeon N Cobbold M Khanna R Nayak L. Herpesvirus-specific CD8 T cell immunity in old age: Cytomegalovirus impairs the response to a coresident EBV infection. J Immunol. (2004) 173:7481–9. doi: 10.4049/jimmunol.173.12.7481 15585874

[29] Moriyama A. Secretory leukocyte protease inhibitor (SLPI) concentrations in cervical mucus of women with normal menstrual cycle. Mol Hum Reproduction. (1999) 5:656–61. doi: 10.1093/molehr/5.7.656 10381821

[30] Wagner B Hillegas JM Babasyan S. Monoclonal antibodies to equine CD23 identify the low-affinity receptor for IgE on subpopulations of IgM+ and IgG1+ B-cells in horses. Veterinary Immunol Immunopathology. (2012) 146:125–34. doi: 10.1016/j.vetimm.2012.02.007 22405681

[31] Schnabel CL Wemette M Babasyan S Freer H Baldwin C Wagner B. C-C motif chemokine ligand (CCL) production in equine peripheral blood mononuclear cells identified by newly generated monoclonal antibodies. Veterinary Immunol Immunopathology. (2018) 204:28–39. doi: 10.1016/j.vetimm.2018.09.003 30596378

[32] Schnabel CL Babasyan S Freer H Wagner B. CXCL10 production in equine monocytes is stimulated by interferon-gamma. Veterinary Immunol Immunopathology. (2019) 207:25–30. doi: 10.1016/j.vetimm.2018.11.016 30593347

[33] Wagner B Hillegas JM Antczak DF. A monoclonal antibody to equine interleukin-4. Veterinary Immunol Immunopathology. (2006) 110:363–7. doi: 10.1016/j.vetimm.2006.01.001 16480777

[34] Larson EM Babasyan S Wagner B. IgE-binding monocytes have an enhanced ability to produce IL-8 (CXCL8) in animals with naturally occurring allergy. JI. (2021) 206:2312–21. doi: 10.4049/jimmunol.2001354 PMC8185406 33952617

[35] Wagner B Radbruch A Rohwer J Leibold W. Monoclonal anti-equine IgE antibodies with specificity for different epitopes on the immunoglobulin heavy chain of native IgE. Veterinary Immunol Immunopathology. (2003) 92:45–60. doi: 10.1016/S0165-2427(03)00007-2 12628763

[36] Kearney JF Radbruch A Liesegang B Rajewsky K. A new mouse myeloma cell line that has lost immunoglobulin expression but permits the construction of antibody-secreting hybrid cell lines. J Immunol. (1979) 123:1548–50. doi: 10.4049/jimmunol.123.4.1548 113458

[37] Wagner B Hillegas JM Brinker DR Horohov DW Antczak DF. Characterization of monoclonal antibodies to equine interleukin-10 and detection of T regulatory 1 cells in horses. Veterinary Immunol Immunopathology. (2008) 122:57–64. doi: 10.1016/j.vetimm.2007.10.012 18061278

[38] Jonsdottir S Svansson V Stefansdottir SB Mäntylä E Marti E Torsteinsdottir S. Oral administration of transgenic barley expressing a *Culicoides* allergen induces specific antibody response. Equine Vet J. (2017) 49:512–8. doi: 10.1111/evj.12655 27859584

[39] Kydd J Antczak DF Allen WR Barbis D Butcher G Davis W. Report of the first international workshop on equine leucocyte antigens, Cambridge, UK, july 1991. Veterinary Immunol Immunopathology. (1994) 42:3–60. doi: 10.1016/0165-2427(94)90088-4 7975180

[40] Lunn DP Holmes MA Antczak DF Agerwal N Baker J Bendali-Ahcene S. Report of the second equine leucocyte antigen workshop, squaw valley, California, july 1995. Veterinary Immunol Immunopathology. (1998) 62:101–43. doi: 10.1016/S0165-2427(97)00160-8 9638857

[41] Sheoran AS Lunn DP Holmes MA. Monoclonal antibodies to subclass-specific antigenic determinants on equine immunoglobulin gamma chains and their characterization. Veterinary Immunol Immunopathology. (1998) 62:153–65. doi: 10.1016/S0165-2427(97)00162-1 9638859

[42] Wagner B Glaser A Hillegas JM Erb H Gold C Freer H. Monoclonal antibodies to equine IgM improve the sensitivity of West Nile virus-specific IgM detection in horses. Veterinary Immunol Immunopathology. (2008) 122:46–56. doi: 10.1016/j.vetimm.2007.10.013 18054390

[43] Kabithe E Hillegas J Stokol T Moore J Wagner B. Monoclonal antibodies to equine CD14. Veterinary Immunol Immunopathology. (2010) 138:149–53. doi: 10.1016/j.vetimm.2010.07.003 20674042

[44] Wagner B Freer H. Development of a bead-based multiplex assay for simultaneous quantification of cytokines in horses. Veterinary Immunol Immunopathology. (2009) 127:242–8. doi: 10.1016/j.vetimm.2008.10.313 19027964

[45] Schnabel CL Babasyan S Freer H Larson EM Wagner B. New mAbs facilitate quantification of secreted equine TNF-α and flow cytometric analysis in monocytes and T cells. Veterinary Immunol Immunopathology. (2021) 238:110284. doi: 10.1016/j.vetimm.2021.110284 34126553

[46] Couto MA Harwig SS Cullor JS Hughes JP Lehrer RI. eNAP-2, a novel cysteine-rich bactericidal peptide from equine leukocytes. Infect Immun. (1992) 60:5042–7. doi: 10.1128/iai.60.12.5042-5047.1992 PMC258275 1452336

[47] Couto MA Harwig SS Lehrer RI. Selective inhibition of microbial serine proteases by eNAP-2, an antimicrobial peptide from equine neutrophils. Infect Immun. (1993) 61:2991–4. doi: 10.1128/iai.61.7.2991-2994.1993 PMC280950 8514405

[48] Tsukishiro S Suzumori N Nishikawa H Arakawa A Suzumori K. Use of serum secretory leukocyte protease inhibitor levels in patients to improve specificity of ovarian cancer diagnosis. Gynecologic Oncol. (2005) 96:516–9. doi: 10.1016/j.ygyno.2004.10.036 15661245

[49] Cummins JE Denniston M Mayer KH Pickard R Novak RM Graham P. Mucosal innate immune factors in secretions from high-risk individuals immunized with a bivalent gp120 vaccine. AIDS Res Hum Retroviruses. (2007) 23:748–54. doi: 10.1089/aid.2006.0233 17531002

[50] Averdunk L Fitzner C Levkovich T Leaf DE Sobotta M Vieten J. Secretory leukocyte protease inhibitor (SLPI)—A novel predictive biomarker of acute kidney injury after cardiac surgery: A prospective observational study. JCM. (2019) 8:1931. doi: 10.3390/jcm8111931 31717603 PMC6912354

[51] Patel RS Tomlinson JE Divers TJ Van De Walle GR Rosenberg BR. Single-cell resolution landscape of equine peripheral blood mononuclear cells reveals diverse cell types including T-bet+ B cells. BMC Biol. (2021) 19:13. doi: 10.1186/s12915-020-00947-5 33482825 PMC7820527

[52] Moreau T Baranger K Dadé S Dallet-Choisy S Guyot N Zani ML. Multifaceted roles of human elafin and secretory leukocyte proteinase inhibitor (SLPI), two serine protease inhibitors of the chelonianin family. Biochimie. (2008) 90:284–95. doi: 10.1016/j.biochi.2007.09.007 17964057

[53] Nugteren S Simons-Oosterhuis Y Menckeberg CL Hulleman-van Haaften DH Lindenbergh-Kortleve DJ Samsom JN. Endogenous secretory leukocyte protease inhibitor inhibits microbial-induced monocyte activation. Eur J Immunol. (2023) 53:2249964. doi: 10.1002/eji.202249964 36480463 PMC10107746

[54] Xu W He B Chiu A Chadburn A Shan M Buldys M. Epithelial cells trigger frontline immunoglobulin class switching through a pathway regulated by the inhibitor SLPI. Nat Immunol. (2007) 8:294–303. doi: 10.1038/ni1434 17259987

[55] Ma G Greenwell-Wild T Lei K Jin W Swisher J Hardegen N. Secretory leukocyte protease inhibitor binds to annexin II, a cofactor for macrophage HIV-1 infection. J Exp Med. (2004) 200:1337–46. doi: 10.1084/jem.20041115 PMC2211913 15545357

[56] Huang Y Wang M Hong Y Bu X Luan G Wang Y. Reduced expression of antimicrobial protein secretory leukoprotease inhibitor and clusterin in chronic rhinosinusitis with nasal polyps. J Immunol Res. (2021) 2021:1–13. doi: 10.1155/2021/1057186 PMC7810533 33506054

[57] Saitoh H Masuda T Shimura S Fushimi T Shirato K. Secretion and gene expression of secretory leukocyte protease inhibitor by human airway submucosal glands. Am J Physiology-Lung Cell Mol Physiol. (2001) 280:L79–87. doi: 10.1152/ajplung.2001.280.1.L79 11133497

[58] Itaoka N Nagamatsu T Schust DJ Ichikawa M Sayama S Iwasawa-Kawai Y. Cervical expression of elafin and SLPI in pregnancy and their association with preterm labor. Am J Reprod Immunol. (2015) 73:536–44. doi: 10.1111/aji.2015.73.issue-6 25559229

[59] Balkus J Agnew K Lawler R Mitchell C Hitti J. Effects of pregnancy and bacterial vaginosis on proinflammatory cytokine and secretory leukocyte protease inhibitor concentrations in vaginal secretions. J Pregnancy. (2010) 2010:1–3. doi: 10.1155/2010/385981 PMC3065848 21490741

[60] Yuan M Jordan F McInnes IB Harnett MM Norman JE. Leukocytes are primed in peripheral blood for activation during term and preterm labour. Mol Hum Reproduction. (2009) 15:713–24. doi: 10.1093/molehr/gap054 PMC2762373 19628509

[61] Griffin JFT Beck I. A longitudinal study of leucocyte numbers and mitogenesis during the last ten weeks of human pregnancy. J Reprod Immunol. (1983) 5:239–47. doi: 10.1016/0165-0378(83)90239-5 6620252

[62] Zhang J Shynlova O Sabra S Bang A Briollais L Lye SJ. Immunophenotyping and activation status of maternal peripheral blood leukocytes during pregnancy and labour, both term and preterm. J Cell Mol Medi. (2017) 21:2386–402. doi: 10.1111/jcmm.13160 PMC5618694 28429508

[63] Bazzano M Giannetto C Fazio F Rizzo M Giudice E Piccione G. Physiological adjustments of haematological profile during the last trimester of pregnancy and the early post partum period in mares. Anim Reprod Science. (2014) 149:199–203. doi: 10.1016/j.anireprosci.2014.07.005 25064559

[64] Blanchard TL Orsini JA Garcia MC Elmore RG Youngquist RS Bierschwal CJ. Influence of dystocia on white blood cell and blood neutrophil counts in mares. Theriogenology. (1986) 25:347–52. doi: 10.1016/0093-691X(86)90070-1 16726126

[65] Aoki T Honda H Ishii M. Immunologic profiles of peripheral blood leukocytes and serum immunoglobulin G concentrations in perinatal mares and neonatal foals (Heavy draft horse). J Equine Veterinary Science. (2013) 33:989–95. doi: 10.1016/j.jevs.2013.03.179

[66] Harvey JW Asquith RL Patel MG Kivipelto J Chen CL Ott EA. Haematological findings in pregnant, postparturient and nursing mares. Comp Haematol Int. (1994) 4:25–9. doi: 10.1007/BF00368262

[67] Gimeno-Molina B Muller I Kropf P Sykes L. The role of neutrophils in pregnancy, term and preterm labour. Life. (2022) 12:1512. doi: 10.3390/life12101512 36294949 PMC9605051

[68] Krohn J Eilenberg RD Gajewski Z Failing K Wehrend A. Lochial and endometrial cytological changes during the first 10 days post-partum with special reference to the nature of foaling and puerperium in equine. Theriogenology. (2019) 139:43–8. doi: 10.1016/j.theriogenology.2019.07.023 31362195

[69] Samejima T Nagamatsu T Akiba N Fujii T Sayama S Kawana K. Secretory leukocyte protease inhibitor and progranulin as possible regulators of cervical remodeling in pregnancy. J Reprod Immunol. (2021) 143:103241. doi: 10.1016/j.jri.2020.103241 33157500

[70] Becquart P Grésenguet G Hocini H Kazatchkine MD Bélec L. Secretory leukocyte protease inhibitor in colostrum and breast milk is not a major determinant of the protection of early postnatal transmission of HIV: AIDS. (1999) 13(18):2599. doi: 10.1097/00002030-199912240-00018 10630534

[71] Si-Tahar M Merlin D Sitaraman S Madara JL. Constitutive and regulated secretion of secretory leukocyte proteinase inhibitor by human intestinal epithelial cells. Gastroenterology. (2000) 118:1061–71. doi: 10.1016/S0016-5085(00)70359-3 10833481

[72] Nyström M Bergenfeldt M Ohlsson K. The elimination of secretory leukocyte protease inhibitor (SLPI) from the gastrointestinal tract in man. Scandinavian J Clin Lab Invest. (1997) 57:119–25. doi: 10.1080/00365519709056379 9200270

[73] Pillay K Coutsoudis A Agadzi-Naqvi AK Kuhn L Coovadia HM Janoff EN. Secretory leukocyte protease inhibitor in vaginal fluids and perinatal human immunodeficiency virus type 1 transmission. J Infect Dis. (2001) 183:653–6. doi: 10.1086/318535 11170993

[74] Jeffcott LB. Passive immunity and its twsfer with special reference to the horse. Biol Rev. (1972) 47:439–64. doi: 10.1111/j.1469-185X.1972.tb01078.x 4574242

[75] Wagner B Flaminio JBF Hillegas J Leibold W Erb HN Antczak DF. Occurrence of IgE in foals: Evidence for transfer of maternal IgE by the colostrum and late onset of endogenous IgE production in the horse. Veterinary Immunol Immunopathology. (2006) 110:269–78. doi: 10.1016/j.vetimm.2005.10.007 16343646

[76] McGuire TC Crawford TB Hallowell AL Macomber LE. Failure of colostral immunoglobulin transfer as an explanation for most infections and deaths of neonatal foals. J Am Vet Med Assoc. (1977) 170:1302–4. 863776

[77] Gensollen T Iyer SS Kasper DL Blumberg RS. How colonization by microbiota in early life shapes the immune system. Science. (2016) 352:539–44. doi: 10.1126/science.aad9378 PMC5050524 27126036

[78] Samsom JN van der Marel APJ van Berkel LA van Helvoort JMLM Simons-Oosterhuis Y Jansen W. Secretory leukoprotease inhibitor in mucosal lymph node dendritic cells regulates the threshold for mucosal tolerance. J Immunol. (2007) 179:6588–95. doi: 10.4049/jimmunol.179.10.6588 17982048

[79] Menckeberg CL Hol J Simons-Oosterhuis Y Raatgeep HRC De Ruiter LF Lindenbergh-Kortleve DJ. Human buccal epithelium acquires microbial hyporesponsiveness at birth, a role for secretory leukocyte protease inhibitor. Gut. (2015) 64:884–93. doi: 10.1136/gutjnl-2013-306149 25056659

[80] Sluis K Darlow B Vissers M Winterbourn C. Proteinase-antiproteinase balance in tracheal aspirates from neonates. Eur Respir J. (1994) 7:251–9. doi: 10.1183/09031936.94.07020251 7909297

[81] Sveger T Ohlsson K Polberger S Noack G Mörse H Laurin S. Tracheobronchial aspirate fluid neutrophil lipocalin, elastase- and neutrophil protease-4-α1-antitrypsin complexes, protease inhibitors and free proteolytic activity in respiratory distress syndrome. Acta Paediatrica. (2007) 91:934–7. doi: 10.1080/080352502760272614 12412868

[82] Watterberg KL Carmichael DF Gerdes JS Werner S Backstrom C Murphy S. Secretory leukocyte protease inhibitor and lung inflammation in developing bronchopulmonary dysplasia. J Pediatrics. (1994) 125:264–9. doi: 10.1016/S0022-3476(94)70209-8 7913723

[83] Harris RA Nagy-Szakal D Mir SA Frank E Szigeti R Kaplan JL. DNA methylation-associated colonic mucosal immune and defense responses in treatment-naïve pediatric ulcerative colitis. Epigenetics. (2014) 9:1131–7. doi: 10.4161/epi.29446 PMC4164498 24937444

[84] Ozaka S Sonoda A Ariki S Kamiyama N Hidano S Sachi N. Protease inhibitory activity of secretory leukocyte protease inhibitor ameliorates murine experimental colitis by protecting the intestinal epithelial barrier. Genes to Cells. (2021) 26:807–22. doi: 10.1111/gtc.12888 34379860

[85] Reardon C Lechmann M Brüstle A Gareau MG Shuman N Philpott D. Thymic stromal lymphopoetin-induced expression of the endogenous inhibitory enzyme SLPI mediates recovery from colonic inflammation. Immunity. (2011) 35:223–35. doi: 10.1016/j.immuni.2011.05.015 PMC3169330 21820333

