# Supplementary material for: Neonatal and maternal upregulation of antileukoproteinase in horses
Source: Front Immunol. 2024 Apr 26;15:1395030. doi: 10.3389/fimmu.2024.1395030 (PMC11082313; doi:10.3389/fimmu.2024.1395030)
Supplement: Supplementary file 2 [file DataSheet_1.pdf]

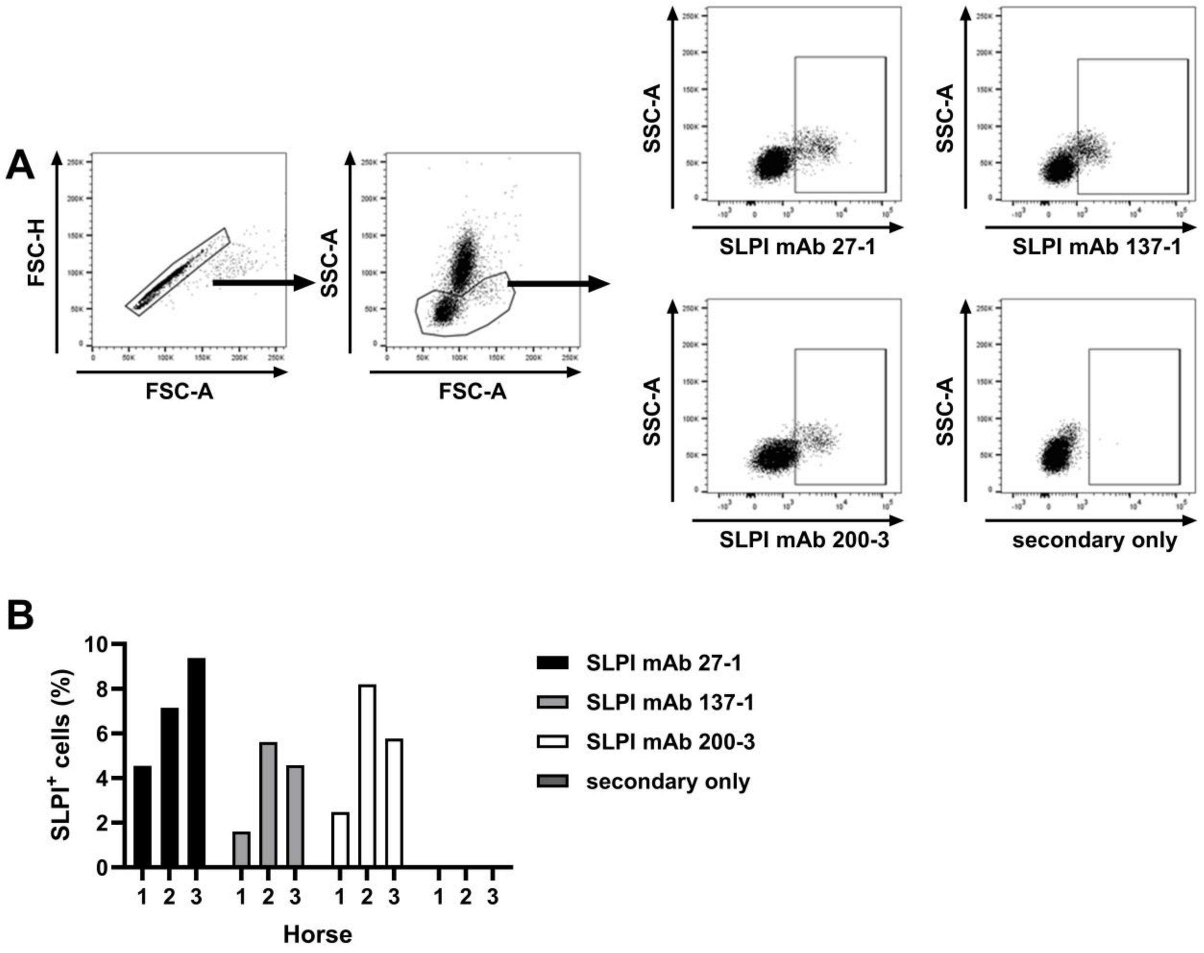

Figure S1: Comparison of three different SLPI mAbs by flow cytometry. PBMC were isolated from healthy horses, fixed, stained intracellularly with one of the SLPI mAbs 27-1, 137-1, or 200-3 and analyzed by flow cytometry. SLPI mAbs were unconjugated and a secondary antibody control staining was included. (A) A representative gating strategy for one horse (Horse 2) is shown. First, a gate was placed on singlets, then on PBMC to analyze SLPI$^+$ cells. (B) The percentages of SLPI$^+$ cells within the total PBMC population are shown for three horses. Each bar represents an individual horse.

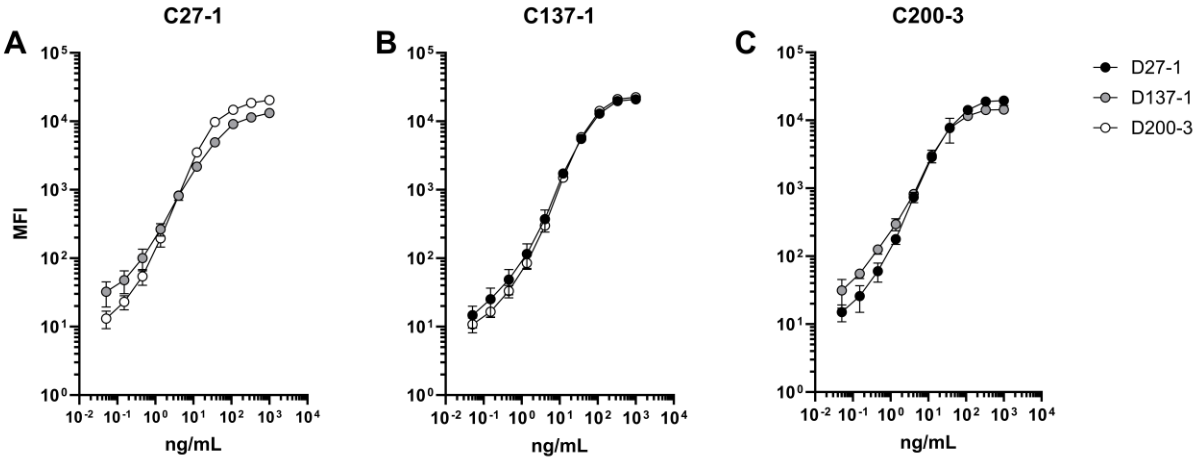

Figure S2: Comparison of mAb pairs for fluorescent bead-based assay. Three newly generated equine SLPI mAbs were tested in different pairing combinations for bead coupling and detection to establish an optimized bead-based assay for quantification of SLPI. The three SLPI mAbs were used for bead coupling: (A) C27-1, (B) C137-1 and (C) C200-3. Beads were incubated with different concentrations of IL-4/SLPI to create standard curves. Afterwards, each assay was detected with biotinylated SLPI mAbs, D27-1 (black), D137-1 (grey), and D200-3 (white), followed by incubation with streptavidin-phycoerythrin. The curves show means and SD from five measurements. MFI = median fluorescent intensity.
